# Intuitive eating is positively associated with indicators of physical and mental health among rural Australian adults

**DOI:** 10.1111/ajr.12856

**Published:** 2022-03-03

**Authors:** Nina Van Dyke, Eric J. Drinkwater

**Affiliations:** ^1^ Mitchell Institute, Victoria University Melbourne Victoria Australia; ^2^ Social Research Centre Melbourne Victoria Australia; ^3^ Centre for Sport Research, School of Exercise & Nutrition Sciences, Deakin University Geelong Victoria Australia; ^4^ School of Allied Health, Exercise and Sports Sciences Charles Sturt University Bathurst New South Wales Australia; ^5^ Present address: Centre for Sport Research School of Exercise & Nutrition Sciences Deakin University Geelong Victoria Australia

**Keywords:** body mass index, intuitive eating, mental health, obesity, self‐esteem

## Abstract

**Introduction:**

Rural Australians have comparatively higher rates of overweight and obesity, as well as some mental health issues. Intuitive eating has been shown to be positively associated with an array of physical and mental health indicators. Few studies, however, have been conducted with general populations, and none has explicitly examined intuitive eating among rural residents.

**Objective:**

To investigate the prevalence of intuitive eating, and associations between intuitive eating and indicators of physical and mental health, among a general population of rural adults.

**Design:**

Cross‐sectional telephone survey of 200 randomly selected, non‐metropolitan, English‐speaking Australian residents aged 18 or older.

**Findings:**

The prevalence of intuitive eating in the sample was 17.6%, with a higher level of intuitive eating among men than women (26.1% vs 9.1%). Bivariate associations between intuitive eating and each of the six health indicators were all positive and mostly statistically significant. Particularly strong was the correlation between intuitive eating and self‐esteem for women (*r* = 0.53). After controlling for indication of an eating disorder and demographics, the associations between intuitive eating and the outcome variables held for body mass index (BMI), psychological distress and body esteem for men, and for BMI and self‐esteem for women. Post hoc analyses found that BMI did not moderate the relationship for women between intuitive eating and self‐esteem and that body esteem mediates the relationships between intuitive eating and BMI and psychological distress for men, and between intuitive eating and self‐esteem for women.

**Discussion:**

Consistent with most prior research, this study finds that intuitive eating is positively associated with several indicators of both physical and mental health among non‐metropolitan residents in Australia. Practice of intuitive eating in this population, however, is low. These findings may help allied health professionals guide rural populations to better health, and may be a particularly effective approach for people for whom the barriers to seeking out health services are high.

**Conclusion:**

Intuitive eating appears to have substantial correlations with mental health indicators, and to some extent, physical health indicators, among rural Australians and therefore should be further investigated for its potential to inform public health policy targeted to similar populations.

**What is already known on this subject:**

- Prior studies have generally found that intuitive eating is correlated with more positive indicators of physical and mental health
- Much of this research, however, consists either of small clinical trials with overweight Caucasian women or surveys conducted with U.S. undergraduate students
- None has explicitly examined rural residents
- Despite higher rates of obesity, self‐harm and suicide among Australian rural residents, no studies have explicitly examined intuitive eating among this population

**What this paper adds:**

- This random, cross‐sectional survey of rural Australian adults provides evidence that intuitive eating in this population is associated with better physical and mental health
- Given this association, along with the low prevalence of intuitive eating in this population, this research suggests that the promotion of intuitive eating might constitute a promising avenue for public health policy for rural residents

## 1 INTRODUCTION

Rural Australians have higher rates of overweight and obesity than those living in metropolitan areas. Results from the 2014–2015 Australian Institute of Health and Welfare study of overweight and obesity rates in Australia show that the rate of overweight or obese adults was 68.5% in regional areas, compared with 60.7% in metropolitan areas.^1^ Obesity and high body mass index (BMI) have been linked to higher mortality rates and diseases such as type 2 diabetes, cardiovascular disease, osteoarthritis and some cancers.^2^ Mental health issues also affect a large percentage of rural Australians.^3^ While overall rates of mental illness are similar for urban and rural residents, rates of self‐harm and suicide increase with remoteness, with significantly higher rates of suicide among rural men.^4^ Moreover, obesity and mental health problems are indirectly related to each other.^5^

Intuitive eating was originally developed by clinicians in response to the poor success of traditional low kilojoule diets in achieving long‐term weight reduction.^6^ The basic tenets of intuitive eating are to respond to innate hunger and satiety signals (i.e. eat when hungry and stop when full, without restrictions on types of food consumed).^7^, ^8^ Research examining the intuitive eating approach finds that it is positively associated with lower BMI and other physical health indicators. Although intuitive eating does not appear to be substantially more successful than low‐calorie dieting for promoting weight *loss*, it might be for weight *maintenance*.^9^ Moreover, a mounting body of evidence indicates that people who eat intuitively have more positive attitudes towards themselves and their bodies.^10^ Much of this research, however, consists either of small clinical trials with overweight Caucasian women or surveys conducted with U.S. undergraduate students.^10^ None has explicitly examined intuitive eating among rural residents.

The purpose of this paper was to examine the prevalence of intuitive eating in a non‐metropolitan population and test associations between intuitive eating and several physical and mental health indicators.

## 2 METHODS

### 2.1 Ethics

Ethics approval was obtained from the Charles Sturt University Human Research Ethics Committee (2010/144).

### 2.2 Participants and procedures

All English‐speaking Australian residents of private dwellings in non‐metropolitan regions, defined as postcodes that correspond to non‐capital city statistical divisions according to the Australian Bureau of Statistics, aged 18 and older with a landline telephone, were eligible to participate in the survey. Two‐hundred people were interviewed by telephone over a two‐week period using computer‐assisted telephone interviewing. To obtain these 200 interviews, 1343 residential numbers were called, and in 742 cases, someone from the household answered the telephone. The interviews were conducted by the Social Research Centre, a social research company. Households were chosen using random digit dialling, with quotas by state or territory corresponding to population percentages. Demographics of respondents are reported in Table 1. Survey respondents were more likely to live in higher population density areas, be female and have a higher level of education as compared to the general population of non‐metropolitan adults.

**TABLE 1:** Demographic and behavioural characteristics of survey participants (*N* = 200) compared with national data

Demographic	Survey participants (%)	Population (2016 ABS census) (%)
ARIA classification^a^		
Inner regional	63.9	42.1
Outer regional	29.5	38.3
Remote	4.2	12.6
Very remote	2.7	7.1
Gender		
Female	57.0	50.6
Male	43.0	49.4
Age		
18–24	7.5	9.8
25–34	10.0	14.6
35–44	18.0	15.6
45–54	28.0	18.0
55–64	19.5	18.3
65–74	12.0	14.6
75+	5.0	9.2
Bachelor's degree or higher	25.5	15.3
Relationship status		
Married or living with a partner	65.0	63.5
Single and not in a relationship	16.5	17.2
Born overseas	19.0	17.5
Household income $80,000+	32.3	46.3
Has health condition that affects weight	23.0	NA
On a diet prescribed by doctor	11.2	NA
Has high blood pressure	23.5	NA
Has high cholesterol	18.0	NA
Smokes daily	15.0	NA
Currently on a weight‐loss diet	8.5	NA
Currently pregnant or breastfeeding	2.5	NA
Intuitive eater^b^	17.6	NA
Men	26.1	NA
Women	9.1	NA

^a^ ARIA classification based on confirmed postcode of participant.

^b^ Mean score of >= 4.0 (‘agree’) on the Tylka IES.

### 2.3 Measures

The following measures of intuitive eating, indication of an eating disorder and indicators of physical and mental health were included in the questionnaire. These measures were reviewed by a panel of experts in the field.

#### 2.3.1 Tylka Intuitive Eating Scale (Tylka IES)

The Tylka IES was used to measure intuitive eating.^7^ This scale comprises three factors: unconditional permission to eat (e.g. ‘I get mad at myself for eating something unhealthy’; ‘I feel guilty if I eat a certain food that is high in calories, fat, or carbohydrates’), eating for physical rather than emotional reasons (e.g. ‘I use food to help me soothe my negative emotions’; ‘I find myself eating when I am stressed out, even when I’m not physically hungry’) and reliance on internal hunger/satiety cues (e.g. ‘When I’m eating, I can tell when I am getting full’; ‘I can tell when I’m slightly hungry’). An ‘intuitive eater’ was defined as anyone who scored an average of 4.0 (‘agree’) on the 5‐point Likert scale ranging from 1 = strongly disagree to 5 = strongly agree, a cut‐off point suggested by Dr. Tylka (email correspondence 28 April 2020). Tylka has subsequently developed the Tylka IES‐2,^8^ a refinement to the Tylka IES; this study was conducted prior to the publication of this revised scale. The original IES (used in this study) has been validated with women in early and middle adulthood^11^ and early adolescents.^12^ The revised IES (IES‐2) has been validated with several general populations around the world.^13^, ^14^

#### 2.3.2 SCOFF

The SCOFF questionnaire^15^ is a screening instrument for detecting eating disorders and was used to measure indication of an eating disorder. It consists of five items with yes–no response options. A score of 2 or greater was originally set as the cut‐off point for maximum sensitivity to detect anorexia and bulimia nervosa.^15^

#### 2.3.3 Body mass index

Body mass index was calculated using self‐reported height and weight. A BMI of >18.5 is considered underweight, between 25 and 30 is categorised as overweight, and over 30 is considered obese.^16^

#### 2.3.4 Blood pressure and cholesterol

Respondents were first asked whether they had had their blood pressure/cholesterol checked by a medical professional within the past two years. If they said they had, they were then asked whether they had high blood pressure/cholesterol or were taking medication to reduce their levels.

#### 2.3.5 Rosenberg Self‐esteem Scale (RSES)

The RSES^17^ was used to measure self‐esteem. The scale consists of 10 items and uses 4‐point Likert scales (from strongly disagree to strongly agree). The items were averaged to obtain a total score. The RSES has been used in Australia in a cross‐national study to explore the universal and culture‐specific features of self‐esteem.^18^

#### 2.3.6 Kessler‐10 (K10)

The K10^19^ was used to measure psychological distress. The 10‐item scale uses 5‐point Likert scales (from all of the time to none of the time). The items were added, yielding a minimum score of 10 and a maximum score of 50, with high scores indicating high levels of psychological distress. The K10 has recently been used with a rural, Australian population.^20^

#### 2.3.7 Body Esteem Scale for adolescents and adults: BE‐appearance (BES‐BE)

The BES‐BE was used to measure body satisfaction.^21^ The scale consists of 10 items and uses 5‐point Likert scales (from none of the time to all of the time). To our knowledge, the BES‐BE has not been used in a study with an Australian adult general population. However, it has been validated among various other populations.^22^, ^23^

### 2.4 Planned analysis

Analyses were conducted using IBM SPSS 26. Preliminary analyses were performed as required to ensure no violations of statistical assumptions (e.g. normality of distribution).

Respondents who reported being on a doctor‐prescribed diet that does not allow them to eat intuitively were omitted from any analyses involving the intuitive eating scale. Descriptive analyses were used to describe the sample. The chi‐squared test for independence and independent‐samples *t* test were used to test gender differences on the main study variables: intuitive eating, BMI, high blood pressure, high cholesterol, self‐esteem, psychological distress and body esteem. Associations between study variables were examined using measures appropriate for continuous (Pearson's product–moment correlation coefficient), ordinal (Spearman rank–order correlation) or dichotomous (point‐biserial correlation coefficient) variables. Differences in correlation coefficients were assessed by Fisher's *r*‐to‐*z* transformation.

Multivariate multiple regression and logistic regression analyses were used to assess the ability of intuitive eating to predict levels of the four continuous dependent variables (BMI, Kessler‐10, RSES and Body Esteem Scale‐BE), and odds ratios for the two dichotomous variables (high blood pressure and high cholesterol), controlling for the influence of indication of an eating disorder, age, education, household income and relationship status (in/not in a relationship).

## 3 RESULTS

### 3.1 Descriptive statistics

Descriptive statistics of the main study variables are presented in Table 2. Omitting data from respondents who are required for medical reasons to follow a diet that prescribes what, when or how much to eat (*n* = 13), survey respondents reported a mean score on the Tylka IES of 3.5, which equates to an average response of between ‘neither agree nor disagree’ and ‘agree’. Omitting respondents who either had a health condition that affects their weight or are pregnant or breastfeeding (*n* = 43), survey respondents had a mean BMI of 26.5 kg/m^2^, indicating that just over half (54.2%) can be considered overweight or obese. Of the 175 respondents who said they had had their blood pressure checked within the past 2 years, 22.3% reported they had high blood pressure; of the 129 respondents who said they had had their cholesterol levels checked within the past 2 years, 22.7% reported they had high cholesterol levels. On average, respondents reported a self‐esteem score of 3.3 on the 4‐point scale, a body esteem score of 3.9 on the 5‐point scale, and a psychological distress score of 16.3 on the 50‐point scale.

**TABLE 2:** Descriptive statistics for key variables

Variable	M	SD	%
Intuitive eating	3.49	0.53	
BMI	26.48	4.96	
High blood pressure			22.3
High cholesterol			22.7
Self‐esteem	3.26	0.46	
Psychological distress	16.32	5.70	
Body esteem	3.93	0.65	

Note

Data are weighted.

### 3.2 Gender differences in intuitive eating and physical and mental health indicators

Comparisons by gender on intuitive eating, BMI, high blood pressure, high cholesterol, self‐esteem, psychological distress and body esteem are presented in Tables 3 and 4. On average, men scored higher on the intuitive eating scale than women did (M = 3.6, SD = 0.54 vs. M = 3.3, SD = 0.49). The only other statistically significant gender difference was for body esteem, in which men, on average, scored higher than women (M = 4.1, SD = 0.56 vs. M = 3.8, SD = 0.67).

**TABLE 3:** Gender differences in intuitive eating, BMI, self‐esteem, psychological distress and body esteem

Measure	Women M (SD)	Men M (SD)	*t*‐value	*p*	*d*
Intuitive eating	3.35 (0.49)	3.64 (0.54)	3.81	<0.001	0.56
BMI	26.26 (5.27)	26.70 (4.63)	1.09	0.171	0.09
Self‐esteem	3.29 (0.47)	3.24 (0.45)	−0.76	0.449	0.11
Psychological distress	16.92 (6.19)	15.68 (5.09)	−1.54	0.126	0.22
Body esteem	3.75 (0.67)	4.12 (0.56)	4.14	<0.001	0.60

**TABLE 4:** Gender differences in high blood pressure and high cholesterol

Measure	Women		Men		Chi^2^(1)	*p*
	*n*	%	*n*	%		
High blood pressure	24	26	15	19	0.86	0.353
High cholesterol	17	23	12	21	<0.001	0.970

### 3.3 Bivariate associations

#### 3.3.1 Intuitive eating and demographic variables

Associations between intuitive eating and the demographic variables are presented in Table 5. Age was associated with intuitive eating for men, with increasing age negatively correlated with intuitive eating, but not for women. A Fisher *r*‐to‐*z* transformation showed these correlations to be different. Household income and intuitive eating had a small/medium‐sized positive correlation for men but not for women, with greater household income associated with higher scores on the intuitive eating scale. No association was found between education and intuitive eating, either for all respondents or when analysed separately by gender. There was also no association between intuitive eating and relationship status (i.e. in a relationship; not in a relationship).

**TABLE 5:** Correlations between intuitive eating and demographic variables

	Age	Household income	Education	Relationship status
Intuitive eating	−0.16*	0.20**	−0.05	0.04
Women	0.06	0.05	0.05	0.16
Men	−0.37***	0.31****	−0.08	−0.10
*p*	0.006	NS	NS	NS

Note

* *p* < 0.05

** *p* < 0.01

*** *p* < 0.001.

#### 3.3.2 Intuitive eating and physical and mental health indicators

Associations between intuitive eating and the three indicators of physical health—BMI, high blood pressure and high cholesterol—and the three indicators of mental health—self‐esteem, psychological distress and body esteem—are presented separately by gender in Table 6. Moderate, negative correlations exist between intuitive eating and BMI for both men and women, with higher scores on the Tylka IES correlated with lower BMI. A moderate, negative association was found between eating intuitively and blood pressure for men but not women, with higher scores on the Tylka IES associated with normal blood pressure. The correlations between intuitive eating and cholesterol levels were not statistically significant for either men or women.

**TABLE 6:** Intercorrelations between intuitive eating and six measures of physical and mental health as a function of gender

Measure	1	2	3	4	5	6	7
1. Intuitive eating	–	−0.37***	−0.29*	−0.21	0.21	−0.29**	0.39***
2. BMI	−0.34**	–	0.44***	0.25	−0.04	0.24*	−0.36**
3. High blood pressure	−0.07	0.24*	–	0.44**	−0.01	0.16	−0.01
4. High cholesterol	−0.00	0.15	0.09	–	−0.26	0.34*	−0.18
5. Self‐esteem	0.53***	−0.03	−0.13	−0.05	–	−0.56***	0.40***
6. Psychological distress	−0.26**	0.04	0.02	0.09	−0.46***	–	−0.44***
7. Body esteem	0.32**	−0.19	−0.22*	−0.13	0.58***	−0.46***	–

Note

Associations for men are presented above the diagonal, and associations for women are presented below the diagonal.

* *p* < 0.05

** *p* < 0.01

*** *p* < 0.001.

Both men and women showed moderate correlations between scores on the intuitive eating scale and psychological distress, with greater intuitive eating associated with less psychological distress. Similarly, there were moderate, positive correlations for both men and women between intuitive eating and body esteem. With self‐esteem, however, there was a large correlation with intuitive eating for women but a non‐significant correlation for men.

### 3.4 Multivariate analyses

Key results of the multivariate analyses are presented in Tables 7 and 8. Higher level of intuitive eating was a statistically significant predictor for both men and women of lower BMI, for men of lower psychological distress and higher body esteem, and for women of higher self‐esteem.

**TABLE 7:** Multivariate multiple regression analysis summary for Tylka IES predicting indicators of physical and mental health, separately by gender

Dependent variables	Men				Women			
	*B*	*SE B*	*t*	*p*	*B*	*SE B*	*t*	*p*
BMI	−2.25	1.00	−2.24	0.028	−4.35	1.15	−3.79	<0.001
RSES	0.14	0.10	1.35	0.182	0.43	0.09	4.73	<0.001
K10	−2.83	1.17	−2.42	0.018	−1.21	1.34	−0.90	0.371
BES‐BE	0.43	0.12	3.58	0.001	0.21	0.15	1.46	0.149

Note

Other independent variables: Age, education, household income, relationship status (in a relationship/not), SCOFF.

**TABLE 8:** Summary of logistic regression analyses—Impact of intuitive eating on high blood pressure and high cholesterol, separately by gender

	DV	*B*	SE	Wald	df	*p*	Odds ratio	95% CI for odds ratio	
								Lower	Upper
Men	BP	−1.30	0.92	2.03	1	0.154	0.27	0.05	1.63
	Chol	−0.90	1.06	0.72	1	0.398	0.41	0.05	3.27
Women	BP	−0.27	0.68	0.16	1	0.693	0.77	0.20	2.88
	Chol	−0.50	0.72	0.48	1	0.490	0.61	0.15	2.48

### 3.5 Post hoc analyses

**FIGURE 1:**
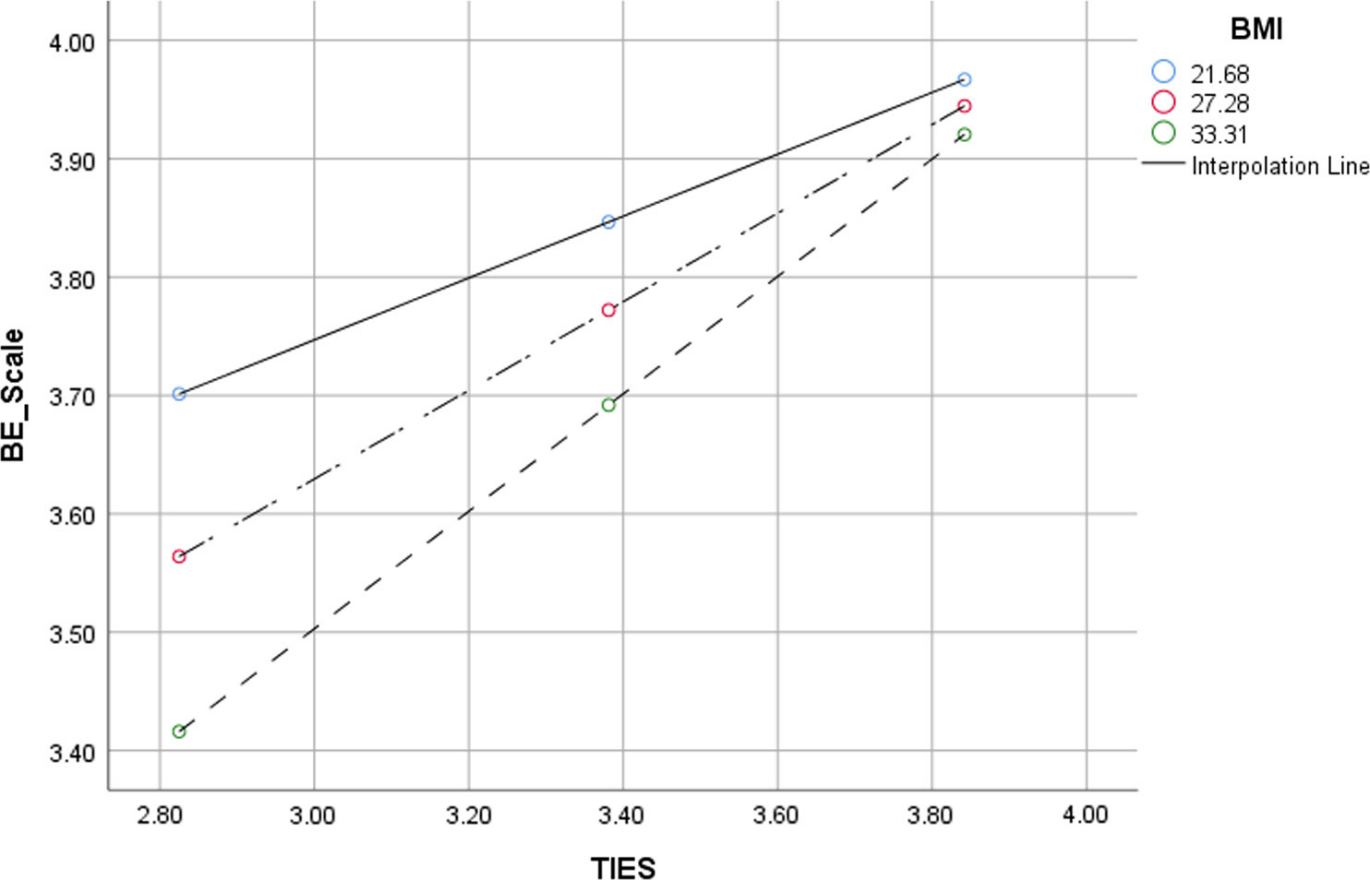
Simple slopes analysis of BMI status as a moderator of the relationship between intuitive eating and body esteem

Given the bivariate association found for women between intuitive eating and body esteem, and prior evidence that level of body esteem varies with BMI,^24^ we conducted a post hoc moderated multiple regression analysis using PROCESS macro v3.3 to test whether BMI moderates the relationship between intuitive eating and body esteem for women in this population. As the multiple regression analysis indicated that intuitive eating was the only independent variable in the model that reached statistical significance, the moderation analysis was run without any covariates. The results are presented in Table 9 and Figure 1.

**TABLE 9:** BMI as a moderator between intuitive eating and body esteem for women

	*b*	*SE B*	*t*	*p*
Constant	3.76 [3.61, 3.92]	0.078	48.14	<0.001
Intuitive eating	0.37 [0.07, 0.68]	0.155	2.42	<0.05
BMI	−0.01 [−0.05, 0.02]	0.016	−0.87	0.386
Intuitive eating x BMI	0.02 [−0.03, 0.07]	0.027	0.74	0.461

Note

*R* ^2^ = 0.10.

The interaction between intuitive eating and BMI was found not to be statistically significant. At low moderation (BMI = 21.7/intuitive eating = 2.82), the conditional effect = 0.2611, 95% C.I. (−0.16, 0.69), *p* = 0.225. At middle moderation (BMI = 27.28/intuitive eating = 3.38), the conditional effect = 0.3742, 95% C.I. (0.07, 0.68), *p* = 0.02. At high moderation (BMI = 33.31/intuitive eating = 3.84), the conditional effect = 0.50, 95% C.I. (0.04, 0.95), *p* = 0.03. These results identify BMI as a non‐moderator of the relationship between intuitive eating and body esteem for women.

In addition, given prior evidence that body esteem might mediate the association between intuitive eating and various outcome variables,^25^ we conducted four simple mediation analyses using PROCESS macro v3.3 to test whether body esteem mediated the relationships between intuitive eating and BMI (men and women), psychological distress (men) and self‐esteem (women), each of which remained statistically significant in the multiple regression analyses. As the multiple regression analyses indicated that intuitive eating was the only independent variable in the BMI (men and women) and psychological distress (men) models that reached statistical significance, these mediation analyses were run without any covariates. In the multiple regression model with self‐esteem as the dependent variable (women), both education and SCOFF, as well as intuitive eating, were statistically significant; therefore, education and SCOFF were included in this mediation analysis as covariates.

The indirect effects of body esteem on BMI for men, psychological distress for men and self‐esteem for women were found to be statistically significant; the indirect effect of body esteem on BMI for women was found not to be statistically significant [BMI (men): effect = −1.8436, 95% C.I. (−4.13, −0.17); BMI (women): effect = −0.2458, 95% C.I. (−0.84, 0.39); K10 (men): effect = −2.1838, 95% C.I. (−5.61, −0.09); RSES (women): effect = 0.0623, 95% C.I. (−0.02, 0.18)].

## 4 DISCUSSION

The aim of this study was to expand the understanding of intuitive eating—both its prevalence and associations with physical and mental health indicators—by focusing on rural residents, a previously ignored population in this literature and one with greater health needs. Consistent with most prior research, this study finds that intuitive eating is positively associated with an array of indicators of both physical and mental health among non‐metropolitan residents in Australia. These indicators include self‐reported BMI, high or normal blood pressure and cholesterol levels, self‐esteem, psychological distress and body esteem. The strength of the associations between intuitive eating and the physical health indicators was mostly small to moderate, with those for BMI for men and women, and blood pressure for men, reaching statistical significance. The associations between intuitive eating and the mental health indicators were mostly larger. Several of these relationships between intuitive eating and the health indicators held after controlling for the SCOFF and demographic variables—BMI for both men and women, psychological distress and body esteem for men, and self‐esteem for women.

In the bivariate analysis, we found a large association between intuitive eating and body esteem for women, although the strength of this association decreased in the multivariate analysis. Body dissatisfaction is a significant psychological issue and is particularly prevalent among women when defined as thinness orientation.^26^ Although one might assume that rural residents, because of their more remote location, would be somewhat protected from body image pressures, the evidence indicates that body image is as pressing an issue in rural areas as elsewhere.^27^ Whereas prior research has found that the strength of the relationship between intuitive eating and body image is moderated by BMI,^26^ our study did not find this effect. Future research might want to explore whether this is due to the nature of the population in this study (rural Australians), or some other reason. The associations between intuitive eating and both psychological distress and self‐esteem are important given the higher levels of some mental health issues in rural areas as compared to metropolitan areas, coupled with lower rates of help‐seeking—due to availability of, travel distance to, and cost of services, as well as attitudinal barriers, such as a disinclination of rural residents to recognise their need for help.^28^

The small associations between intuitive eating and physical health indicators are likely due to higher quality nutritional intake, which results in lower weight and thus BMI; lower BMI then might positively affect blood pressure and cholesterol levels. Research findings on intuitive eating and dietary intake are somewhat mixed, but most suggest a positive association.^29^ Rural residents, however, due to low population size and remoteness, have less access to a wide variety of healthy foods.^30^

The prevalence of intuitive eating among rural Australian adults is low, however, at just over 9% of women and 26% of men. It is difficult to know how this result compares with levels of intuitive eating among other populations as different measures have been used in different studies. For example, a study of young people from 31 metropolitan area middle and high schools in Minnesota found that among young adult men, 74.8% indicated that they trusted their body to tell them how much to eat, and 79.1% reported that they stopped eating when full. Among young adult women, 64.8% indicated that they trusted their body to tell them how much to eat, and 76.4% reported that they stopped eating when full.^31^ In our study, we considered an ‘intuitive eater’ as anyone with an average score of 4 or higher on the entire intuitive eating scale, a definition suggested by the developer of the intuitive eating scale.^7^

Several of the findings from this study have implications for health promotion programs seeking to promote healthier eating and for mental health literacy. The finding that intuitive eating is more common among men, and particularly younger men and men with higher household incomes, for example, suggests that different targeted messages or approaches might be necessary for women, older men and men living in household with lower incomes.

Future research should investigate why the prevalence of intuitive eating is low and consider possible ways to increase this percentage. Higher rates of intuitive eating in men than in women, as well as the finding of a negative association for men but not for women between intuitive eating and age, have been reported elsewhere in non‐rural populations.^32^ Perhaps as men partner and have children, coupled with the fact that women continue to do most of the family meal preparation, including in rural families,^33^ men's control over what and when they eat and thus their levels of intuitive eating drop. Moreover, as men age, they might also become more concerned about health issues and, in response, attempt to change their previously innate eating patterns. In addition, since young women are generally more concerned about their weight than are young men,^26^ women might veer from eating intuitively in an attempt to reduce their body mass by controlling their diet at a younger age than do men. An issue not addressed by this research, but which future studies might want to explore, is how easy or difficult it is for people who live in rural areas to eat intuitively. The circumstances and considerations around choices of what, when and how much to eat are complex, perhaps made more difficult for rural residents by their more remote location.^34^

While additional research is needed on the causal relationship between intuitive eating and improved health, particularly in the longer term,^35^ and the ease or difficulty with which it can be adopted and practised, these findings might help allied health professionals guide rural populations to better health, and may be a particularly effective approach for people for whom the barriers to seeking out health services are high.

### 4.1 Strengths and limitations

A strength of this study is the use of random sampling to gather data from a general population. In addition, whereas some prior studies have included only one or two health indicators (e.g. BMI), or focused on only physical health indicators or mental health indicators, this study includes three indicators of physical health and three indicators of mental health. Limitations include the relatively small sample size (*n* = 200) and low response rate (1343 numbers dialled to obtain 200 survey completions), self‐reporting of physical and mental health and cross‐sectional design, which does not allow for determination of causality.

This study fills an important gap in the growing evidence base around intuitive eating by examining rural residents, who have different health needs and barriers, and who thus far have largely been ignored in the intuitive eating literature. This research provides evidence that intuitive eating is associated with better physical and mental health among non‐metropolitan Australian adults and thus constitutes a promising avenue for current public health policy in this area.

## CONFLICT OF INTEREST

Neither Dr Van Dyke nor Dr Drinkwater has any conflict of interest to disclose.

## AUTHOR CONTRIBUTION

NVD: conceptualization; formal analysis; investigation; methodology; writing – original draft. EJD: conceptualization; formal analysis; funding acquisition; writing – original draft; writing – review & editing.

## 5 Ethics approval

Charles Sturt University (2010/144).
